# Patients’ preferences, experiences and expectations with wait time until surgery in gynaecological oncology: a mixed-methods study in two gynaecological oncological centres in the Netherlands

**DOI:** 10.1136/bmjopen-2024-085932

**Published:** 2024-08-17

**Authors:** Kim van den Berg, Anne Knegt, Guus Fons, Christianne A R Lok, Johanna W M Aarts

**Affiliations:** 1 Amsterdam UMC Locatie De Boelelaan, Amsterdam, The Netherlands; 2 Flevoziekenhuis, Almere, Netherlands; 3 Centre for Gynaecologic Oncology Amsterdam, Amsterdam, Netherlands; 4 Gynaecologic Oncology, Antoni van Leeuwenhoek Netherlands Cancer Institute, Amsterdam, Netherlands

**Keywords:** Patient-Centered Care, Gynaecological oncology, Organisation of health services

## Abstract

**Abstract:**

**Objectives:**

Patient-centredness of care during wait time before surgery can be improved. In this study we aimed to assess (1) patients’ experiences with and preferences regarding wait time before surgery; (2) the impact of wait time on quality of life (QoL) and (3) which factors influence patients’ wait time experience.

**Design, setting, participants:**

We performed an exploratory sequential mixed-methods study among women with gynaecological cancer in two tertiary hospitals. We conducted semistructured interviews and identified aspects of QoL and factors that influenced wait time acceptability through thematic analysis. We developed a questionnaire from this thematic analysis which was completed by 97 women. Descriptive statistics and univariate and multivariate regression analyses were performed.

**Results:**

Average ideal wait time was 3.5 weeks (±1.7 weeks), minimum and maximum acceptable wait times were 2.2 and 5.6 weeks. Many patients scored above the threshold of the Hospital Anxiety and Depression Scale for anxiety (48%) or depression (34%), had sleeping problems (56%) or experienced pain (54%). A number of factors were more common in patients who indicated that their wait time had been too long: low education level (OR 7.4, 95% CI 0.5 to 5.0, p=0.007), time to surgery >4 weeks (OR 7.0, 95% CI 0.8 to 4.4, p=0.002) and experienced sleep disturbance (OR 3.27, 95% CI 0.0 to 3.1, p=0.05). If patients expectation of wait time was >4 weeks (OR 0.20, 95% CI −4.0 to −0.5 p=0008) or if patients experienced pain (OR 0.26, 95% CI −3.6 to −0.3, p=0.03), they less frequently indicated that wait time had been too long.

**Conclusion:**

To improve patient-centredness of care, healthcare providers should aim to reduce wait time to 3–4 weeks and ensure that patients are well informed about the length of wait time and are aware of high levels of anxiety, depression and pain during this time. Future studies should evaluate what interventions can improve QoL during wait time.

**STRENGTHS AND LIMITATIONS OF THIS STUDY**

- The mixed-methods research design allowed us to combine the strengths of qualitative and quantitative data, enriching quantitative results with semistructured interviews and validating the qualitative findings with the results from 97 respondents to the questionnaire.
- Methodology of paper with interviews and questionnaire means that the patient’s perspective is the centre of this research.
- The cross-sectional design does not allow us to draw conclusions on causality.
- Our population may not be representative of all populations, around 35% had a high educational level and we had few participants with a non-Dutch ethnicity.

## Introduction

 Patient-centredness is an important dimension of quality of care. There are multiple dimensions to patient-centred care. The Institute of Medicine endorses six dimensions, including care respectful to patients’ values, preferences and expressed needs,^1^ to help us define patient-centred care. Healthcare organisations increasingly focus on integrating patients’ preferences and needs into the delivery of healthcare, and they guide delivery by their patients’ values.^2^ Providing patient-centred care has several presumed benefits, such as increasing the patient’s quality of life (QoL). Most studies in gynaecological cancer care have focused on improving patient-centredness of care during or after treatment^3 4^ while the wait time period before surgery has been neglected.

Timeliness of care is increasingly an issue of concern for both patients and providers. Incidences of cancer are rising, in the Netherlands, around 58 000 people received a diagnosis in 1990 compared with nearly 129 000 in 2023.^5^ With limited resources, such as a lack of sufficient qualified staff and financial restraints, it is a big challenge for healthcare providers to provide high-quality care to the increasing number of people diagnosed with cancer in the coming years. During the SARS-COV-2 pandemic, wait times before surgery increased significantly^6^ and are likely to further increase as a result of these challenges. Increased wait times before surgery due to SARS-COV-2 pandemic were associated with increased anxiety and depression in women with cancer.^7^ As care providers, we need to know how patients experience wait time and how care could be more in line with their preferences and expectations.

Most women diagnosed with gynaecological cancer will undergo surgery as part of their treatment. They will almost always have to wait at least sometime before surgery can take place. Current literature and guidelines state that, for most gynaecological cancers, treatment has to start within 28–31 days after the decision on the treatment plan.^8^,^11^ In the Netherlands, the Foundation for Interdisciplinary Knowledge-Sharing and -Development (SONCOS in Dutch) formulates maximum wait times, similar to for instance the British Gynaecological Cancer Society. This wait time is justified as it has no impact on prognosis.^10^,^14^ However, it is not known how wait time relates to other outcomes. For instance, in other types of cancer, longer wait time is associated with high levels of psychological distress and anxiety.^15^,^18^ Moreover, when asking patients, they prefer a shorter wait time to surgery,^19^,^22^ and in a discrete choice experiment, patients with gynaecological cancer were willing to pay more if that would lead to wait time reduction.^23^

Knowing patients’ preferences and experiences about wait time can improve patient-centredness of care, as has been shown in studies for instance in the field of reproductive medicine.^24 25^ Furthermore, it can help healthcare services set goals for maximum wait times and help healthcare providers to support patients during wait time. Ultimately, more patient-centred care might improve patient’s QoL during wait time, which may also lead to better QoL after treatment.^26^

Studies on QoL postsurgery in gynaecological oncology showed that many aspects of QoL are affected for an extended period of time.^27 28^ The aim of this study was, therefore, to evaluate (1) patients’ experiences with and preferences regarding current wait times before surgery for gynaecological cancer; (2) the impact of wait times on the QoL and (3) which factors influence patients’ experiences with wait time.

## Methods

We performed an exploratory sequential mixed-methods study among women with a (suspected) gynaecological cancer in two tertiary hospitals in Amsterdam, the Netherlands. As the literature on this topic is scarce, we used qualitative methods to gain initial insights into the patient’s perspective on wait time (qualitative part). Consequently, we used these qualitative data to develop a questionnaire to validate these findings for a larger number of people. To that end, a larger cross-sectional quantitative study was performed (quantitative part).

### Setting, study population and definitions

In the Netherlands, gynaecological oncological care is centralised and provided in nine oncological centres. Patients diagnosed with gynaecological cancer are referred to one of these centres. This study took place in two gynaecological oncological centres, providing care to almost 25% of the Dutch population. The study population consisted of women who underwent surgery for (suspected) gynaecological cancer, that is, vulvar, endometrial, ovarian and cervical cancer. The types of surgery women underwent were staging laparoscopy or laparotomy, radical hysterectomy with pelvic node dissection, primary debulking surgery, exploratory laparotomy with or without frozen section, radical vulvectomy with or without sentinel node procedure or inguinal lymph node dissection. For both the qualitative and quantitative part of the study, we included patients during their postoperative hospital stay to limit recall bias. We excluded patients who underwent neoadjuvant treatment and were scheduled for interval debulking surgery, patients who underwent diagnostic or very small procedures and did not require an overnight stay and women who were not able to speak English or Dutch for the interviews. For the questionnaires, women with a language or literacy barrier were not excluded if someone could help them fill out the questionnaire. Women who underwent exploratory surgery because of suspected cancer but final pathology showed a benign tumour, were also eligible for inclusion.

### Definition wait time

**Figure 1:**
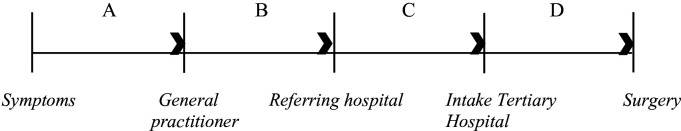
Overview of the different phases patients go through from symptoms through surgery.

In this study, two separate wait times were defined: the first as the time between the referral from a referring hospital to the tertiary hospital (C in figure 1) and the second as the time between the first appointment in the tertiary hospital and the date of surgery (D in figure 1).

### Patient and public involvement

There was no direct patient and public involvement in the design of this study.

### Part A qualitative part: semistructured interviews

#### Data collection and analyses

An interview guide was constructed based on the existing literature and the expertise of the authors. The interview guide was pilot-tested with two patients to ensure that the line of questions would provide the insights we were aiming for. Eligible patients were identified in the week prior to admission based on surgical date and type and approached by their primary physicians during admission. Informed consent was obtained and patients were interviewed during hospital admission, after their surgery. We conducted 16 semistructured interviews until no new themes emerged. Interviews were recorded, transcribed verbatim and analysed using thematic analysis. Two researchers (KvdB and MP) coded the interviews independently and organised all relevant statements in themes. This was discussed with a third researcher (JWMA) until a consensus was reached. These themes were used to develop the questionnaire. Online supplemental table 1 gives an overview of the link between the themes derived from the interviews and the questionnaire (online supplemental file 3). The interview guide is added as online supplemental file 2.

#### Development of questionnaire

Based on the themes resulting from the qualitative part, a questionnaire was developed consisting of several parts. Part 1 included background characteristics of patients, such as age and educational level. Part 2 aimed at identifying actual wait times, patients’ opinions on these wait times. We also asked them to report ideal wait time; the time they would ideally choose to wait if they could personally determine wait time and minimum and maximum acceptable wait times. In part 3, patients were asked to rate the importance of wait time relative to other aspects of care. Part 4 consisted of questions about physical and emotional well-being during wait time including Hospital Anxiety and Depression Scale (HADS). The HADS is composed of two scales—anxiety (seven items) and depression (seven items). Items are rated on 4-point Likert scale and range from 0 to 3, with higher scores indicating greater anxiety and depression, respectively. The two scores were calculated separately and classified as normal (score 0–7), borderline^8^,^10^ and disturbance (>11).^29^ The HADS has been validated for use in the Netherlands.^30^ In addition, we asked about the impact of time spent on various activities during wait time. Online supplemental table 1 includes an overview of the types of questions for all these parts and how they link to the semistructured interviews. To test the face validity of the questionnaire, it was pilot-tested with a few patients.

### Part B: quantitative part: cross-sectional study using the questionnaire

#### Data collection

The questionnaire was administered to patients admitted for surgery in the period between December 2021 and October 2022. Patients were approached by their lead physicians during admission or by researchers based on screening of eligibility on surgical date and type. If patients were eligible and gave informed consent, they were given a questionnaire during hospital stay. Patients filled out the questionnaire during admission or at home shortly after discharge using a return envelope. The following data were collected from the electronic health record: actual wait times between date of referral, date of first appointment and date of surgery; type of cancer and International Federation of Gynaecology and Obstetrics stage of disease; type of surgery; distance to the hospital from patient’s home (straight line measurements). All data were entered in Data in SPSS V.28.0.1.1.

#### Data analysis

If participants did not fill out a part of the questionnaire completely, that part was excluded from analyses. We used descriptive statistics to present background characteristics (part 1). Descriptive statistics were also used to present actual, ideal and acceptable wait times (part 2). We used descriptive statistics to analyse how patients rank the importance of wait time compared with other aspects of care (part 3). In part 3, patients were asked to rank their top five most important care aspects out of the 11 themes that were derived from the interviews. We calculated how often participants ranked these various aspects. To analyse the impact of wait time before surgery on various aspects of QoL, we used descriptive statistics (part 4). We performed univariate and multivariate regression analysis to determine which factors were associated with patient’s opinions on experienced wait time.

##### Dependent variable

The dependent variable was patients’ opinion on experienced wait time; a dichotomous variable as one of the options ‘too short’ was never given (options were: wait time was ‘too long’, ‘exactly right’ or ‘too short’. A logistic regression analysis was used.

##### Independent variables

As independent variables, we used patients’ background characteristics, type and stage of the disease, type of surgery, if malignant diagnosis was known prior to surgery and if disease was recurrent. Other independent variables were the wait time patients expected before surgery, the time that patients actual waited and how important wait time is to patients. Finally, the presence of pain, pain score during wait time, the presence of sleeping problems and other physical symptoms, anxiety score and depression score (based on HADS) and reduction of time spent working were also used as independent variables.

##### Analysis

Pearson correlation tests were used to rule out collinearity between independent variables. If a correlation between two variables was more than 0.6, we excluded one from further analysis after discussion within the research team on which item was clinically most relevant or theoretically likely to be correlated to the dependent variable. Subsequently, we performed bivariate regression analyses for each of the independent variables with the two different dependent variables, that is, patient’s ideal wait time and patient’s opinion about experienced wait time. We included the variables with a p<0.2 in the multivariate regression analysis. For multivariate analysis, we used a backward selection method. We considered a p<0.05 to be statistically significant. We calculated the odds, p values and 95% CIs. To measure the explained variance of the two models, we used the R^2^ coefficient, indicating what percentage of variance attributed to the patients’ opinion on wait time.

## Results

### Part A: thematic analysis of interviews (qualitative part)

We conducted semistructured interviews with 16 women. Mean age was 56.9 years. Four underwent exploratory laparotomy for adnexal mass, four had ovarian cancer, three had endometrial cancer, four had vulvar cancer and one had cervical cancer. The thematic analysis of the transcripts of the interviews resulted in a total of 61 codes. These codes were then grouped into (sub)themes and finally into four overarching themes.

#### Theme 1: wait time

Two subthemes emerged, that is, duration of wait time and the patient’s opinion of wait time.

All patients were able to give some indication of how long they waited before surgery. Patients used different appointments as reference points for wait time. ‘They referred me and then I had an appointment, I think a few days later’. Many people indicated some sort of opinion on duration of wait time. Patients mentioned that referral time was quite quick ‘And I had my first appointment January 15th. That was quite quick’. Four patients indicated that wait time until surgery had been too long, one that it was too short and five that it had been acceptable. ‘It is always too long’ said one patient whereas another patient indicated ‘I thought it was a bit too quick’, mentioning requiring sometime to prepare. Maximum acceptable wait time varied between 2 and 6 weeks.

#### Theme 2: importance of wait time

Wait time was important to patients ‘it cannot take too long’. Other aspects of care that were mentioned were clear information, clarity about the date of surgery ;‘To know when I am going [to be operated on]. To know the next step, that is important to me’, detailed information about treatment ‘In a very clear way, it [the surgical plan] was also drawn’. Furthermore patients mentioned easily reached care providers ‘I didn’t have to do anything, didn’t have to arrange appointments. I was called all the time. Very well arranged. That is important’, consistency of care providers ‘The man (treating physician) really took care of me during the whole process’ and sufficient attention to physical ‘it was hurting so much that they changed the procedure to be under general anaesthesia’ state. Patients also indicated appreciating attention to emotional state and expectations as well as reliability ‘he did not call until after Easter’ and possibilities to set schedule by patients.

#### Theme 3: factors that impacted wait time acceptability

Importance of short wait time was linked to prognosis ‘because it is an illness that has to come out’, pain ‘when you are in pain, you want to go as quickly as possible’ and ability to participate in essential activities such as caring for others or work ‘if you have to take care of a baby, you want to go as quickly as possible’. Patients identified personality differences ‘I am a positive person, with a sense of humour and I think what has to happen, happens’, prior expectations, and relationship with both referring and treating physicians as factors that impact wait time acceptability. Other items mentioned were familiarity with hospital and pandemic constraints.

#### Theme 4: aspects of life impacted during wait time

Participants mentioned that their lives were greatly impacted by the diagnosis during wait time and that the impact was on various dimensions. ‘It is very emotional, that you get this diagnosis’. They mentioned impact on before surgery on various dimensions: family life ‘my children came regularly, my son with bags full of fruit for smoothies’ and personal relationships ‘much more contact with people. Talking, calling on the phone’ as well as the ability to be active. Emotional well-being ‘I was so sad and scared’, physical well-being ‘pain kept getting worse, and pressure on my bladder’, sleeping ‘I slept so much’ and the ability to work (both paid and volunteer work) were often mentioned. Many, though not all, impacts were described as negative. Some participants mentioned the diagnosis being a turning point for improvement of their lifestyle.

### Part B: questionnaire results (quantitative part)

A total of 134 questionnaires were handed out and 97 were returned (response rate 72%). One patient was excluded because she underwent interval debulking surgery. Table 1 gives an overview of background characteristics.

**Table 1:** Baseline characteristics of all participating patients

	Value	All patients
		n=96
Demographic information		
Age (years), mean±SD		61.3±14.7
Ethnicity, n (%)	Dutch	88 (91.7)
	Other	8 (8.3)
Educational level, n (%)	High school or less	36 (37.5)
	Vocational	25 (26.05)
	Higher vocational or university	34 (35.5)
	Unknown	1 (1.0)
	Single	7 (7.3)
Marital state, n (%)	Married or cohabiting	65 (67.7)
	Widowed	13 (13.5)
	Divorced	9 (9.4)
	Living apart together	2 (2.1)
Children at home n (%) <17 years old, n (%)		16 (16.7)
Distance to the hospital in km, n (%)	0–10 km	21 (21.9)
	10–50 km	48 (50.0)
	>50 km	27 (28.1)
Clinical information		
Patients with recurrent disease, n (%)		15 (15.6)
Diagnosis presurgery, n (%)	Ovarian cancer	17 (17.7)
	Endometrial cancer	21 (21.9)
	Vulvar cancer	22 (22.9)
	Cervical cancer	14 (14.6)
	Suspected cancer	22 (21.9)
Stage after surgery, n (%)	Undefined*	5 (5.2)
	Stage I	39 (40.6)
	Stage II	8 (8.3)
	Stage III	17 (17.7)
	Stage IV	4 (4.2)
	Benign	11 (11.5)
	Borderline tumour ovary	5 (5.2)
	Metastasised non-gynaecological tumour†	1 (1.0)
	Not appplicable‡	5 (5.2)
Type of surgery, n (%)	Radical hysterectomy with PLND	11 (11.5)
	Laparoscopic salpingo-oophorectomy	2 (2.1)
	Staging surgery	8 (8.2)
	Primary debulking surgery	14 (14.6)
	Abdominal hysterectomy	7 (7.3)
	Exploratory laparotomy	20 (20.8)
	Total laparoscopic or robot assisted hysterectomy	8 (8.4)
	WLE/Radical vulvectomy	21 (21.9)
	Robot-assisted staging surgery	5 (5.2)

Mean ± standard deviation or number in the group is shown. Abbreviations: PLND = pelvic lymph node dissection; WLE=Wide local excision.

* Stage was classified as undefined if there was insufficient information after surgery for classification of FIGO stage.

† Krukenberg tumour

‡ Consists of recurrent disease without proper staging afterwards

### Actual wait time and preferences regarding wait time

**Figure 2:**
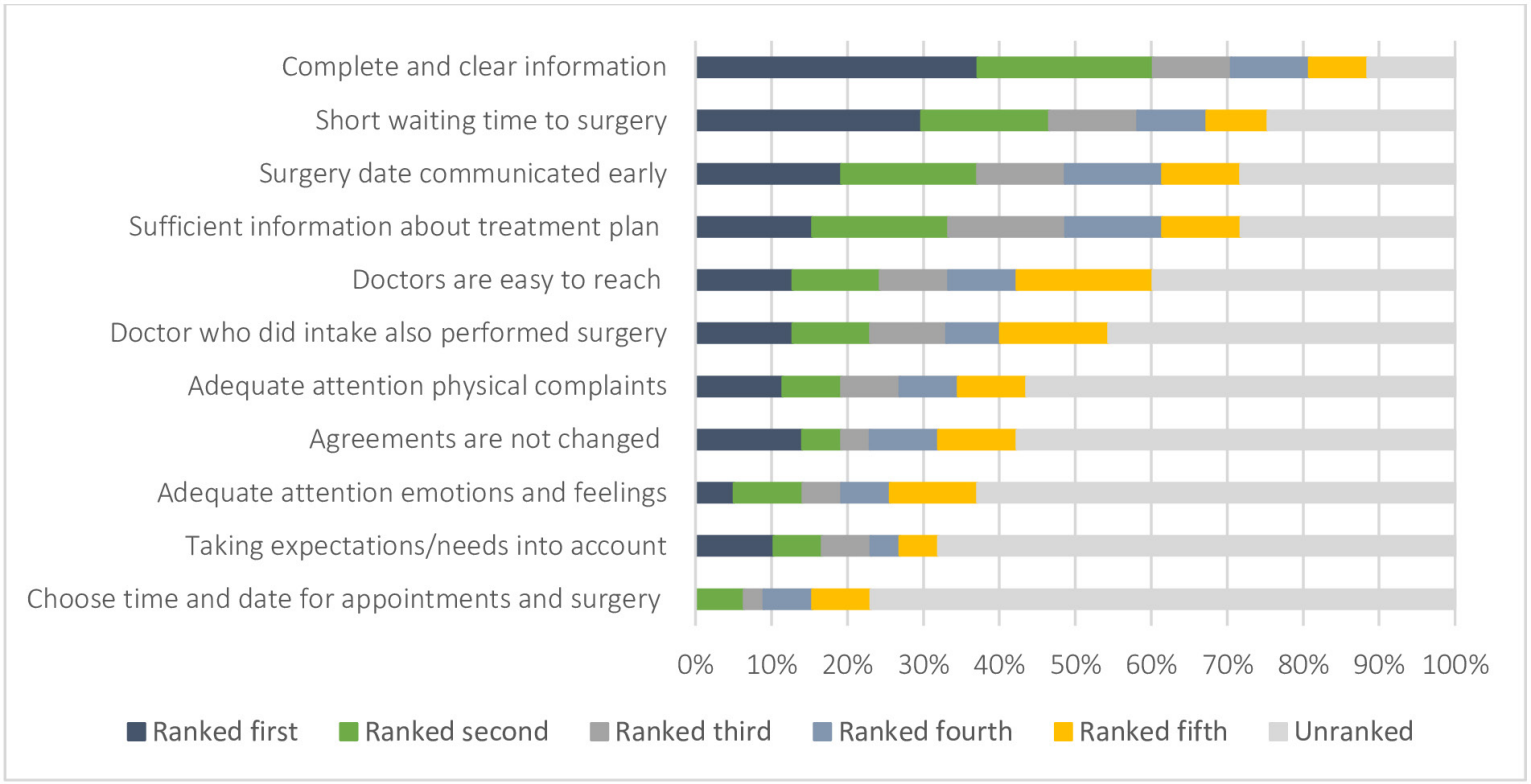
Relative importance of different aspects of care: percentage of patients who ranked an aspect of care as first through fifth most important.

Mean wait time between referral and first consultation was 12 days (±7.0 SD), that is, 1.7 weeks. The mean wait time between the first consultation and surgery was 32 days (±13.2 SD) or 4.6 weeks and 49 patients (51.0%) reported waiting over 4 weeks before surgery. Mean patient-reported ideal wait time to surgery was 3.5 weeks (±1.7 SD), that is, 24.5 days. The mean minimal and maximal acceptable wait time was 2.2 weeks (±1.7 SD), that is, 15.4 days and 5.6 weeks (±5.9 SD), that is, 39.2 days, respectively. In relation to other care aspects, wait time was the second most important (figure 2). The most important was complete and clear information about diagnosis, treatment, admission procedures and realistic information about wait times to surgery. Figure 2 shows how patients ranked importance of different aspects of care during wait time.

### Impact of wait time on QoL

Table 2 shows the number of patients who experienced anxiety, depression, pain and changes in sleeping pattern during wait time.

**Table 2:** Impact on quality of life during wait time (N=96)

Symptoms	Categories	Frequencies n (%)
Anxiety	No anxiety (HADS 0–7)	48 (50.0)
	Possible anxiety disorder (HADS 8–10)	14 (14.6)
	Suspected anxiety disorder (HADS>11)	32 (33.3)
	Missing	2 (2.1)
Depression	No depression (HADS 0–8)	61 (63.5)
	Possible depression (HADS 8–10)	16 (16.7)
	Suspected depression (HADS>11)	17 (17.7)
	Missing	2 (2.1)
Experienced pain	Yes	52 (54.2)
	No	40 (41.7)
	Missing	4 (4.2)
VAS score if women experienced pain (n=52)^a^	General, mean±SD	5.3±2.6 SD
	Maximum, mean±SD	6.1±2.9 SD
Sleeping pattern, n (%)	As much as before	42 (43.8)
	A little less than before	30 (31.3)
	A lot less than before or required medication	24 (25.0)
	Missing	0

Pain score on visual analogue scale from 0 (no pain) to 10 (worst conceivable pain)

HADSHospital Anxiety and Depression ScaleVASVisual Analogue Scale

Patients reported spending less time doing sports or physical activities (41.7%), such as housekeeping (35.4%) during wait time. They reported spending more of their time searching the internet about the disease (38.5%), relaxing (36.5%) or being with family and friends (26.0%). Prior to diagnosis, 42.7% of patients had paid employment, 11.5% did voluntary work and 3.1% did both. Of these patients, 33% stopped working completely during wait time. Reasons for stopping or working less were emotional burden (n=16), physical complaints (n=15) or advice from their (occupational health) doctor (n=8).

### Factors related to preferred wait time and opinion on experienced wait time

Table 3 shows the outcome of the multivariate regression analysis. Patients with a lower educational level (compared with intermediate or high), with a wait time over 4 weeks and with sleeping problems more often indicated that their wait time had been too long. Patients who expected a wait time of more than 4 weeks, less often reported their wait time to be too long. The same accounted for patients who reported experiencing pain. The estimation of the explained variance of our predicting model was R^2^=0.31.

**Table 3:** Multivariate relationship of determinants of the opinion of wait time too long

Independent variable	Coefficient	OR	95% CI		P-value
			LL	UL	
Intercept	−0.632	0.531			0.426
Educational level					0.010
Low	2.001	7.395	0.503	4.954	0.007
Intermediate	0.044	1.004	0.350	5.008	0.679
Time to surgery (>4 weeks)	1.951	7.037	0.799	4.423	0.002
Expected time to surgery (>4 weeks)	−1.635	0.195	−4.006	−0.545	0.008
Presence of pain (yes)	−1.343	0.261	−3.632	−0.258	0.033
Sleep problems (yes)	1.184	3.268	0.027	3.125	0.049S

LLLower limit of 95% confidence interval ULUpper Limit of 95% Confidence Interval

## Discussion

In this study, we demonstrated that patients preferred a wait time between first consultation and surgery for gynaecological cancer of around 3.5 weeks, with minimum and maximum wait times of 2.2 and 5.6 weeks respectively. In reality, 51% of patients waited more than 4 weeks. Multiple aspects of QoL of patients were affected during wait time: from their daily activities to experiencing symptoms of anxiety, depression and pain. The patient’s expectation of wait time appeared to be an important factor in their experiences with wait time.

Based on our findings, we see a number of ways in which patient-centredness of care regarding wait time can be improved. The first way is related to the duration of wait time. Patients in our study preferred wait times to be limited to around 3–4 weeks. Patients preferred some wait time; from the interviews, we gathered that they need sometime to prepare themselves for surgery, both mentally and practically. We also found that our participants preferred a shorter wait time compared with the 4–8 week limits based on studies on wait times for gynaecological cancers with a focus on oncological outcomes, such as survival.^5^,^9^ A goal of 3–4 weeks may not always be realistic especially given current challenges with staff shortages in the healthcare sector increasing demand due to ageing of population and financial and other resource constraints. If there are no additional care providers, surgical time, money and other resources, it would imply that other patients would have to wait longer. A number of studies demonstrated that patients with other diseases such as cataract surgery, hip and knee replacement and coronary artery bypass grafting also prefer shorter wait times^14^,^17^ and that severity of symptoms is associated with a lower tolerability of waiting.^16^ We have not found any studies that directly compare wait time preference between patients with cancer and patients with other diseases which means that it is unclear whether we should prioritise patients with cancer over other diseases beyond the limits set by impact on disease progression. During interviews, many women mentioned that their concerns about possible disease progression determined mostly by their wish for less wait time. Eliciting the reason for the preference of short wait time and giving patients adequate information about the (lack of) impact of waiting on disease outcome may thus be beneficial to reduce distress. Our study emphasised that managing patients’ expectations is very important. First, we showed that if patients expected to wait over 4 weeks prior to their first consultation, their ideal wait time was longer and they less frequently reported having waited too long themselves. Second, receiving complete and correct information about diagnosis, treatment and planning was the most important aspect of care, in line with other research.^31^,^33^ In summary, to improve patient-centredness of care, we recommend to limit wait time ideally to 3–4 weeks. Moreover, we recommend to provide clear upfront information about what patients can expect, ideally given prior referral on both the expected length of wait time and what wait time is known to be safe without having an effect on disease outcome.

The second way to improve patient-centredness of care is management of those aspects of QoL that are negatively impacted during wait time. Mental and physical well-being and thereby QoL are relatively low during wait time, being in line with studies done on the impact of wait times in other types of cancer.^10^,^13^ We found high rates of anxiety (33%) and depression (17%). Although QoL during wait time for patients with gynaecological cancer has not been evaluated before, these percentages are higher than in more general studies on QoL among patients with gynaecological cancer (14%–15% for anxiety and 5.5%–6% for depression).^34 35^ The high prevalence in this study might be explained by the fact that our patients are mostly newly diagnosed patients with cancer and have active disease, all factors related to higher levels of emotional distress.^36^ In the interviews worry about progression of disease or the strong feeling that the disease needed to be removed from the body came up often. It may also mean that wait time is a particularly stressful period of time in the whole treatment. Given that patients tend to under-report symptoms and physicians tend to underestimate severity of distress and impact on QoL.^37 38^ It is very likely that overall we underestimate this problem in daily practice.

In short, the lack of research on patients’ preferences and experiences in this important and stressful period of time, makes it very difficult to define and thereby organise patient-centred care. We have filled part of that knowledge gap. We recommend that screening for anxiety and depression and providing psychosocial support and counselling when needed, is offered during wait time. This has been shown to be beneficial.^39^ The same holds for pain screening and offering adequate pain medication. We also recommend to implement (e-Health) programmes to help patients improve their pre-and postoperative physical and mental well-being.

### Strengths and limitations

The methodology of this paper with semistructured interviews followed by questionnaire results allows a focus on patients’ perspectives. Interviews were conducted until data saturation was reached and the study size for the questionnaire was sufficiently large to result in representative results.

A limitation is the cross-sectional design, which does not allow us to draw conclusions on causality. Second, the data of our study are self-reported and subject to recall bias. The outcome of the surgery, especially in cases where there was uncertainty about the diagnosis presurgery may have influenced results. Our population is not representative of all populations, around 35% had a high education level and we had few participants with a non-Dutch ethnicity.

### Future directions

Further research should focus on which interventions will help patients cope better with both physical and emotional distress during wait time. Specifically how to implement screening for anxiety and depression already prior to surgery and improved methods of allowing patients to signal they experience symptoms such as pain or sleeping issues is recommended. This research should ideally include the longer-term impact of these interventions. In addition, studies on healthcare efficiency and how to improve wait times may give guidance on how to reduce wait times. Finally, research on improving patient education on how to prepare for surgery and how to change the wait time from a relatively passive to a more participatory period seems worthwhile.

## Conclusion

Overall, this study has demonstrated that there are a number of ways to improve patient-centredness of care during wait time. Ideally, wait time should be limited to 3–4 weeks. Given resource (financial and personnel) constraints, this may not always be feasible, but patients should be informed about the length of wait time. If expectations on wait time are in line with actual wait times, patients less frequently report finding their wait time too long. In addition, care providers should be aware that anxiety, depression, sleeping disturbance and pain are common during wait time. Improving patient-centredness of care may thus imply that screening for these conditions can help identify patients who are in need of further support. Finally, we think that more research is warranted to analyse which interventions can help improve QoL during wait time.

## supplementary material

10.1136/bmjopen-2024-085932 online supplemental file 1

10.1136/bmjopen-2024-085932 online supplemental file 2

10.1136/bmjopen-2024-085932 online supplemental file 3

## Data Availability

Data are available on reasonable request.
