# Supplementary material for: Patients’ preferences, experiences and expectations with wait time until surgery in gynaecological oncology: a mixed-methods study in two gynaecological oncological centres in the Netherlands
Source: BMJ Open. 2024 Aug 17;14(8):e085932. doi: 10.1136/bmjopen-2024-085932 (PMC11331850; doi:10.1136/bmjopen-2024-085932)
Supplement: online supplemental file 1 [file bmjopen-14-8-s001.pdf]

Supplementary Table 1 Schematic overview of questionnaire

	Interviews	Topic	Questionnaire
Part 1		Demographics	Age, marital status, children at home, educational level, ethnicity
Part 2	Definition of wait time<br>Minimum and maximum wait time	Duration of and opinion on wait time	Duration of own wait times<br>Opinion on own wait time<br>Minimally and maximally acceptable wait times
Part 3	Factors that impact wait time acceptability: expectations, trust, distance to hospital, physician preference	Relative importance of wait time compared to other aspects of care	Ranking 11 factors that impact quality of care
Part 4	Factors that impact wait time acceptability: Emotional distress, pain, severity of illness<br><br>Impact of wait time on various dimensions : family life, activities, working, sleeping, pain, emotional well being.	Quality of life and activities during wait time	HADS questionnaire<br>Sleeping pattern<br>Pain scores<br>Physical complaints<br>Time spent on activities during wait time
