# Supplementary material for: Patients’ preferences, experiences and expectations with wait time until surgery in gynaecological oncology: a mixed-methods study in two gynaecological oncological centres in the Netherlands
Source: BMJ Open. 2024 Aug 17;14(8):e085932. doi: 10.1136/bmjopen-2024-085932 (PMC11331850; doi:10.1136/bmjopen-2024-085932)
Supplement: online supplemental file 2 [file bmjopen-14-8-s002.pdf]

Interview guide semi-gestructureerde interviews

Voorstellen van de onderzoeker die het interview uit voert.

In dit onderzoek willen we graag inzicht krijgen in de ervaringen die u hebt met de wachttijd tot aan de operatie. Het interview duurt ongeveer 30 minuten. Om de resultaten goed te kunnen analyseren zouden we het interview graag opnemen. Vindt u dat goed?

[zo ja, dan dit antwoord ook nog opnemen op het bandje]

- Kunt u kort vertellen wat voor soort afspraken u heeft gehad in het ziekenhuis en wanneer deze waren? Dus hoe het is gelopen sinds u door de huisarts bent door verwezen naar het ziekenhuis?
  Doorvragen:
  - Wanneer hebt u uw eerste bezoek gehad aan de gynaecoloog?
  - Wanneer bent u voor het eerst hier in het AMC gekomen?
  - Wanneer hoorde u uw diagnose (indien van toepassing; natuurlijk niet bij proeflap)?
  - Hoe lang van tevoren hoorde u de operatiedatum?

- Wat heeft u de afgelopen weken overdag gedaan?
  - Zagen de dagen er anders uit dan voordat u voor het eerst bij de huisarts was geweest?
    - Zo ja vanaf wanneer zagen ze er anders uit?

  Voorbeeld doorvragen
  - Hebt u in die tijd contact opgenomen met de huisarts of het ziekenhuis? Zo ja, welke reden had dit? (klachten/onrust/angst)
  - Bent u nog aan het werk? Net zoveel als daarvoor? Gedurende de hele periode? Bij minder werk: financiële gevolgen?
  - Hobby's?
  - Tijd met familie / gezin?
  - Lichamelijke inspanning?

- **Vragen over de ervaring van patiënten met de wachttijd tot de ingreep.**
  De ervaringen worden specifieker uitgevraagd naar de dimensies van patiëntgerichtheid van zorg (bron: Picker institute)
  1. Respect voor patient warden, voorkeuren en behoeften
  2. Coordinatie en integratie van zorg
  3. Informatievoorziening, communicatie en voorlichting
  4. Fysiek comfort
  5. Emotionele steun en verlichting van angst
  6. Betrokkenheid van familie en vrienden
  7. Continuiteit van zorg
  8. Toegankelijkheid van zorg

- Als u denkt aan de tijd sinds uw eerste bezoek aan de gynaecoloog en de operatiedatum: hoe hebt u deze tijd ervaren?

- Hoe hebt u de tijd tot aan de operatie ervaren?
  Voorbeeld doorvragen
    - Fysiek? Toename klachten tijdens wachten? Effect klachten op functioneren?
    - Emotioneel / psychisch? Bij angst/stressklachten doorvragen op inhoud en effect op functioneren. Beloop emotionele en psychische klachten over tijd: toename of verandering klachten voor en na bepaling van de operatiedatum?
    - Effect op partner/ geliefden
    - Zijn hier nog aparte periodes in te herkennen?

- Zijn er dingen die de wachttijd tot de operatie makkelijker/beter hadden gemaakt?
    - Goede communicatie / informatie voorziening bijvoorbeeld?
    - Duidelijk wie het aanspreekpunt was?
    - Duidelijk wat de stappen waren en waarom er soms wachttijd was?
    - Emotionele ondersteuning?
    - Voldoende eigen invloed op de operatiedatum?

- Hoe lang zou er maximaal aan tijd moeten zitten volgens u tussen
    - Het horen van de diagnose en de operatie?
    - Het horen van het behandelplan en de operatie?

- Wanneer zou u een langere wachttijd tot de operatie acceptabel vinden?

- Zijn er andere zaken die voor u belangrijk zijn omtrent de operatie?

  Voorbeeld doorvragen:

    - Afstand tot ziekenhuis?
    - Mogelijkheid tot ontvangen bezoek in het ziekenhuis
    - Eigen gynaecoloog ook operateur?
