# Supplementary material for: Patients’ preferences, experiences and expectations with wait time until surgery in gynaecological oncology: a mixed-methods study in two gynaecological oncological centres in the Netherlands
Source: BMJ Open. 2024 Aug 17;14(8):e085932. doi: 10.1136/bmjopen-2024-085932 (PMC11331850; doi:10.1136/bmjopen-2024-085932)
Supplement: online supplemental file 3 [file bmjopen-14-8-s003.pdf]

Studienummer ________________________

Datum: ___ / ___ / ___

Vragenlijst voor deelname aan onderzoek

Onderzoek naar ervaringen van patiënten met de wachttijd tot een operatie

Beste mevrouw,

Hartelijk dank voor het meewerken aan dit onderzoek. We zijn geïnteresseerd in wat het wachten op een operatie voor u heeft betekend. We willen graag weten hoe die wachttijd er voor u heeft uitgezien. We willen beter begrijpen wat de invloed van wachten op u is en wat u belangrijk vindt in die wachttijd. Zo willen we kijken of en waar we de zorg moeten aanpassen om deze beter op uw verwachtingen en wensen aan te laten sluiten.

In deze vragenlijst hebben we het steeds over ‘wachttijd’. Deze wachttijd bestaat uit verschillende stappen. Zo heeft u waarschijnlijk moeten wachten tot uw eerste afspraak in het ziekenhuis en na deze afspraak weer tot u geopereerd werd. Voor iedereen kunnen de stappen waaruit deze wachttijd bestaat anders zijn. Hieronder staat als voorbeeld een tijdsbalk met de stappen A tot en met D waar uw wachttijd mogelijk uit bestaan heeft. Het kan dat uw traject er iets anders uitzag. Dat is geen probleem voor het invullen van deze vragenlijst.

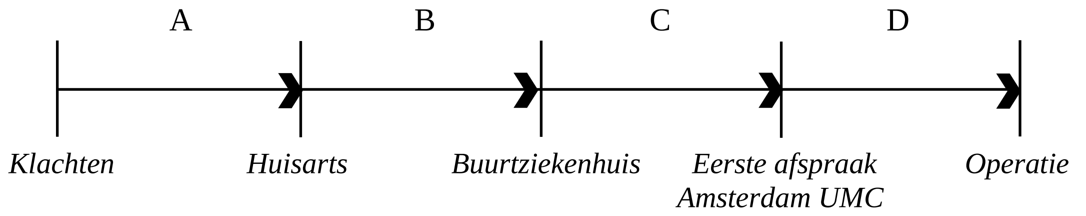

Vanaf het moment dat u klachten heeft tot uw uiteindelijk operatie zijn er de volgende (mogelijke) stappen. Tussen al deze stappen kan er tijd hebben gezeten, die onderdeel zijn van de wachttijd:

A. *U had klachten en besluit naar de huisarts te gaan. U wacht op de afspraak bij de huisarts.*
B. *De huisarts stuurt u door naar een buurtziekenhuis. U wacht op de afspraak in het ziekenhuis.*
C. *In het buurtziekenhuis wordt onderzoek uitgevoerd. U krijgt te horen dat er (mogelijk) sprake is van kanker. U wordt door het buurtziekenhuis doorgestuurd naar het Amsterdam UMC. U wacht op uw eerste afspraak in het Amsterdam UMC. De dokters kijken naar alle informatie en maken met u een plan voor de behandeling.*
D. *Een onderdeel van het plan voor uw behandeling is dat u een operatie zal krijgen. U wacht tot de operatie.*

De vragen die in deze vragenlijst gesteld worden, gaan over verschillende delen van deze 'wachttijd'. Er zal telkens duidelijk aangegeven worden voor welke periode u antwoord moet geven.

De vragenlijst bestaat uit vier delen. Voor elk onderdeel staat een korte uitleg. Lees deze uitleg rustig door en beantwoord vervolgens de daaropvolgende vragen. De meeste vragen kunt u beantwoorden door het antwoord dat voor u 'juist' is te omcirkelen. Indien iets anders gewenst is, zal dit bij de vraag duidelijk worden aangegeven. Er zijn geen 'goede' of 'slechte' antwoorden, dus kies het antwoord dat voor u het meest van toepassing is.

Het invullen van de vragenlijst zal ongeveer 25 minuten duren.

Onderdeel 1:

In dit onderdeel willen we wat algemene vragen over u stellen.

1. Uw leeftijd is _____ jaar.

2. Wat is uw burgerlijke staat?

   A. Alleenstaand
   B. Getrouwd
   C. Weduwe
   D. Gescheiden
   E. LAT relatie
   F. Overig:_________________

3. Hoeveel kinderen van 17 of jonger wonen er bij u thuis? _____________________

4. Uw hoogst behaalde opleiding is:

   A. Lager dan middelbareschooldiploma
   B. Middelbare school diploma of vergelijkbaar
   C. Middelbaar beroepsonderwijs
   D. Hoger beroepsonderwijs
   E. Wetenschappelijk onderwijs

5. Hoe zou u uw etniciteit beschrijven:
   a. Nederlandse
   b. Antilliaanse
   c. Surinaamse
   d. Turkse
   e. Marokkaanse
   f. Anders, namelijk:_____________________

AMC
Meibergdreef 9
1105 AZ Amsterdam
Postbus 22660
1100 DD Amsterdam
T +31(0)20 566 9111
www.amc.nl

Onderdeel 2:

In dit onderdeel willen we graag weten hoe lang uw wachttijd was en of u dit (te) lang of (te) kort vond of precies goed. Ook vragen we naar hoe lang er volgens u minimaal én maximaal aan tijd zou moeten zitten tussen de eerste afspraak in het Amsterdam UMC en de operatie. Omcirkel het antwoord dat voor u van toepassing is. We gebruiken hier dezelfde 'stappen' in de wachttijd als in het voorbeeld in de begintekst. Bij elke vraag is aangegeven over welke stap het gaat.

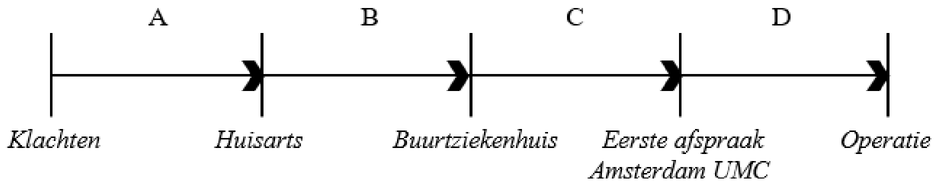

1. Hoeveel tijd zat er tussen het moment dat u werd doorgestuurd door het buurtziekenhuis en de eerste afspraak in het Amsterdam UMC (stap C in het voorbeeld)?

   A. Korter dan 1 week
   B. Tussen 1 en 2 weken
   C. Langer dan 2 weken
   D. Niet van toepassing

2. Wat vond u van deze wachttijd?

   A. Te lang
   B. Precies goed
   C. Te kort

3. Hoeveel tijd zat er tussen de eerste afspraak in het Amsterdam UMC en de operatie (stap D in het voorbeeld)?

   A. Korter dan 1 week
   B. Tussen 1 en 2 weken
   C. Tussen 2 en 3 weken
   D. Tussen 3 en 4 weken
   E. Langer dan 4 weken

4. Wat vond u van deze wachttijd?

   A. Te lang
   B. Precies goed
   C. Te kort

AMC
Meibergdreef 9
1105 AZ Amsterdam
Postbus 22660
1100 DD Amsterdam
T +31(0)20 566 9111
www.amc.nl

5. Toen u in het buurtziekenhuis te horen kreeg dat u geopereerd zou worden, is u toen verteld hoe lang u zou moeten wachten op deze operatie (stap C en D in het voorbeeld)?

   (Bij 'nee' of 'niet van toepassing' als antwoord, ga direct naar vraag 7)

   A. Ja
   B. Nee
   C. Niet van toepassing

6. Hoe lang zou u volgens de artsen in het buurtziekenhuis moeten wachten op de operatie (stap C en D in het voorbeeld)?

   A. Tussen 0 en 2 weken
   B. Tussen 2 en 4 weken
   C. Tussen 4 en 6 weken
   D. Tussen 6 en 8 weken
   E. Langer dan 8 weken

7. Hoe lang had u verwacht te moeten wachten op de operatie vóórdat u in het Amsterdam UMC te horen kreeg hoe lang u zou moeten wachten?

   A. Tussen 0 en 2 weken
   B. Tussen 2 en 4 weken
   C. Tussen 4 en 6 weken
   D. Tussen 6 en 8 weken
   E. Langer dan 8 weken

8. Hoe lang voor de operatie kreeg u de operatiedatum te horen?

   A. Tussen 1 en 3 dagen voor de operatie
   B. Tussen 4 en 7 dagen voor de operatie
   C. Tussen 7 en 14 dagen voor de operatie
   D. Langer dan 14 dagen voor de operatie

9. Wat vond u van de tijd tussen het horen van de operatiedatum en de operatie zelf?

   A. Te lang
   B. Precies goed
   C. Te kort

10. Geef bij het plaatje op de volgende pagina aan hoeveel weken wachten op de operatie voor u acceptabel zou zijn. Het gaat dan om de tijd tussen de eerste afspraak in het Amsterdam UMC en de operatie (stap D in het voorbeeld). U kleurt hiervoor het gebied in tussen het aantal weken wachten dat u <u>minimaal acceptabel</u> vindt en het aantal weken dat u <u>maximaal acceptabel</u> vindt.

Bijvoorbeeld: u vindt zelf dat vanaf de eerste afspraak in dit ziekenhuis minimaal 8 weken wachten en maximaal 11 weken tot de operatie fijn is. U <u>kleurt</u> dan het gebied tussen de 8 en de 11 <u>in</u>. Zoals hier:

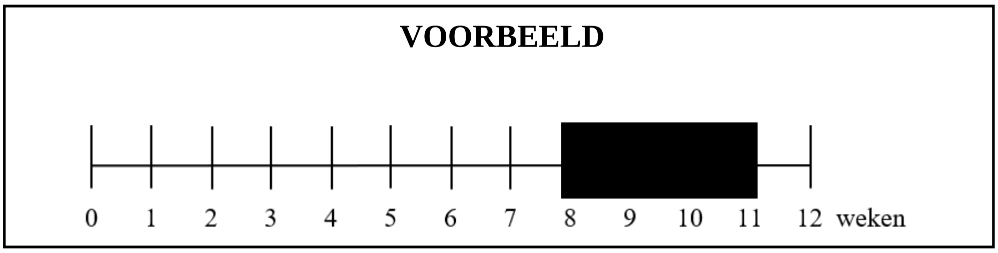

<u>Kleur nu zelf in</u> hoeveel weken wachten op de operatie voor u redelijk zou zijn:

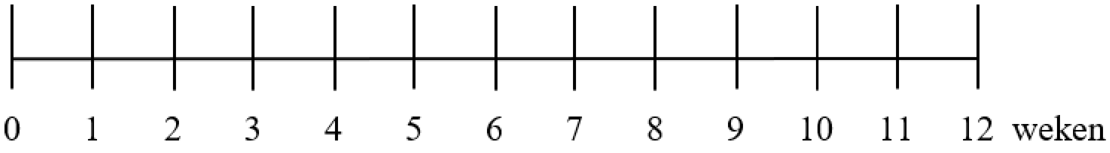

11. Omcirkel op het plaatje het aantal weken wachten dat voor u <u>precies goed</u> zou zijn tussen de eerste afspraak in het Amsterdam UMC en de operatie (stap D in het voorbeeld).

Bijvoorbeeld: u vindt zelf een wachttijd van 10 weken tussen de eerste afspraak in dit ziekenhuis en de operatie perfect. U <u>omcirkelt</u> dan in de figuur het cijfer 10. Zoals hier:

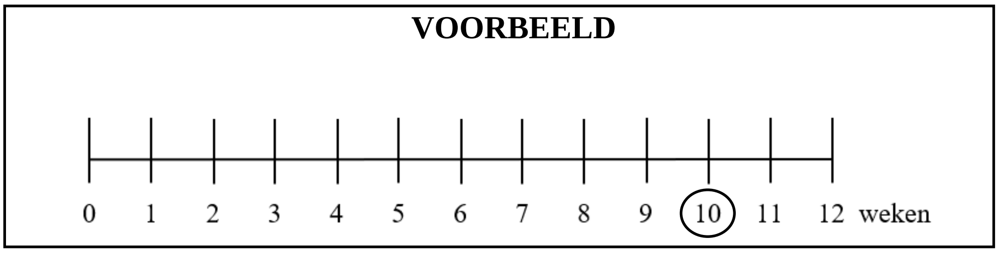

<u>Omcirkel</u> nu zelf het aantal weken wachten dat voor u precies goed zou zijn:

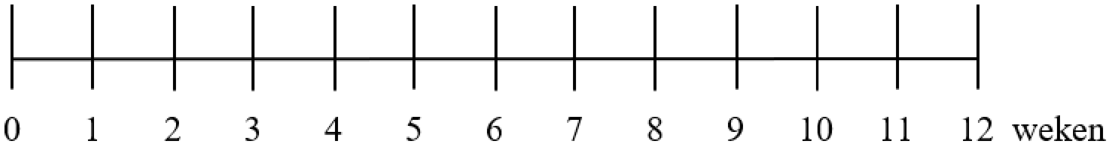

AMC
Meibergdreef 9
1105 AZ Amsterdam
Postbus 22660
1100 DD Amsterdam
T +31(0)20 566 9111
www.amc.nl

12. Als u kijkt naar de mogelijke stappen in de wachttijd: welke stap zou het MINST lang moeten duren?

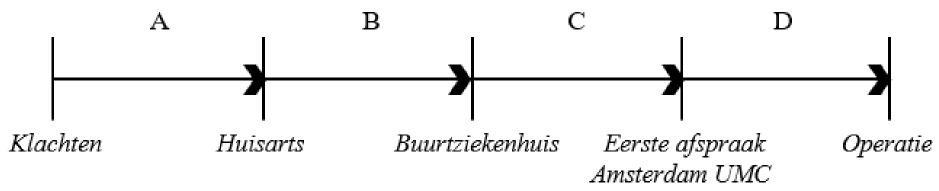

De wachttijd zou het MINST lang moeten zijn tussen:

A. Het hebben van klachten en de afspraak bij de huisarts
B. De verwijzing vanuit de huisarts naar het buurtziekenhuis
C. De verwijzing vanuit het buurtziekenhuis naar het Amsterdam UMC
D. De eerste afspraak in het Amsterdam UMC en de operatie
E. Niet van toepassing

Geef van onderstaande twee uitspraken aan in hoeverre u het ermee eens bent:

13. Patiënten die veel pijn hebben, moeten eerder geopereerd te worden. Zelfs als dat betekent dat ik dan later aan de beurt ben.

A. Helemaal mee eens
B. Enigszins mee eens
C. Enigszins oneens
D. Oneens

14. Patiënten die in de wachttijd moeten stoppen met werken, moeten eerder geopereerd worden. Zelfs als ik dan later aan de beurt ben.

A. Helemaal mee eens
B. Enigszins mee eens
C. Enigszins oneens
D. Oneens

15. De belangrijkste reden om eerder aan de beurt te zijn bij een operatie is voor mij:

(Hier mag u zelf aangeven wanneer mensen volgens u voorrang zouden mogen krijgen bij het krijgen van een operatie)

____

AMC
Meibergdreef 9
1105 AZ Amsterdam
Postbus 22660
1100 DD Amsterdam
T +31(0)20 566 9111
www.amc.nl

Onderdeel 3:

In dit onderdeel willen we graag van u weten of er dingen zijn waardoor u langer zou willen wachten op een operatie. Misschien wilt u bijvoorbeeld wel langer wachten als u dan dichter bij huis geopereerd kan worden. Het kan ook zijn dat een zo kort mogelijke wachttijd voor u het allerbelangrijkste is.

Hieronder ziet u een aantal kenmerken die te maken hebben met het ziekenhuis zelf. Geef hieronder aan wat voor u belangrijker en minder belangrijk is als u nadenkt over in welk ziekenhuis u geopereerd wordt. Vul daarvoor de getallen 1 tot en met 5 in de vakjes achter de uitspraken. Geef daarvoor wat u het ALLERBELANGRIJKST vindt een 1 en wat U het MINST BELANGRIJK vindt een 5.

Het ziekenhuis ligt dichtbij uw woonplaats.	
U bent reeds bekend in het ziekenhuis.	
Het ziekenhuis staat er om bekend goede zorg te leveren.	
De huisarts of verwijzer geeft aan dat dit ziekenhuis de beste optie voor u is.	
Het ziekenhuis heeft de kortste wachttijd.	

We willen graag weten wat voor u belangrijk is in de zorg die u krijgt van het ziekenhuis tijdens de tijd die u moet wachten op een operatie. Hieronder staan vragen die gaan over de manier waarop het ziekenhuis voor u gezorgd heeft in de wachttijd. Kruis het antwoord aan dat aangeeft hoe belangrijk een bepaald deel van deze zorg voor u is. Vul dit in voor de tijd tussen de eerste afspraak in het Amsterdam UMC en de operatie (stap D in het voorbeeld).

We willen graag weten wat in het algemeen belangrijk voor u is en dus niet per se wat uw ervaring is geweest. Als zorgverleners bijvoorbeeld goed rekening hebben gehouden met uw wensen (vraag 1), maar u vindt dit niet zo belangrijk dan vragen we u te antwoorden dat u dit niet zo belangrijk vindt.

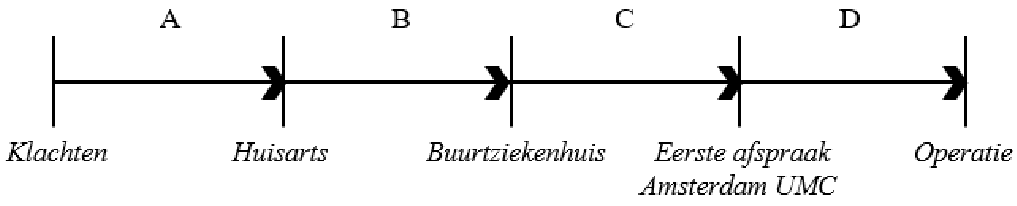

<u>Wat vindt u belangrijk in de wachttijd tot een operatie?</u>

1. De zorgverleners houden rekening met uw wensen en verwachtingen.

○	○	○	○	○
Onbelangrijk	Enigszins onbelangrijk	Neutraal	Enigszins belangrijk	Erg belangrijk

2. U heeft de afspraken in het ziekenhuis tijdens de wachttijd én de operatie bij steeds dezelfde arts.

○	○	○	○	○
Onbelangrijk	Enigszins onbelangrijk	Neutraal	Enigszins belangrijk	Erg belangrijk

3. De informatie over de ziekte, afspraken en operatie die u krijgt tijdens de wachttijd is compleet en duidelijk.

○	○	○	○	○
Onbelangrijk	Enigszins onbelangrijk	Neutraal	Enigszins belangrijk	Erg belangrijk

4. De operatiedatum is snel bekend.

○	○	○	○	○
Onbelangrijk	Enigszins onbelangrijk	Neutraal	Enigszins belangrijk	Erg belangrijk

AMC
Meibergdreef 9
1105 AZ Amsterdam
Postbus 22660
1100 DD Amsterdam
T +31(0)20 566 9111
www.amc.nl

5. Er is voldoende aandacht voor lichamelijke klachten tijdens de wachttijd.

○	○	○	○	○
Onbelangrijk	Enigszins onbelangrijk	Neutraal	Enigszins belangrijk	Erg belangrijk

6. Er is voldoende aandacht voor uw gevoelens en emoties tijdens de wachttijd.

○	○	○	○	○
Onbelangrijk	Enigszins onbelangrijk	Neutraal	Enigszins belangrijk	Erg belangrijk

7. U krijgt voldoende informatie over wat de volgende stappen in de behandeling zijn.

○	○	○	○	○
Onbelangrijk	Enigszins onbelangrijk	Neutraal	Enigszins belangrijk	Erg belangrijk

8. Het ziekenhuis houdt zich aan gemaakte afspraken en deze worden niet meer veranderd.

○	○	○	○	○
Onbelangrijk	Enigszins onbelangrijk	Neutraal	Enigszins belangrijk	Erg belangrijk

9. U kunt zelf aangeven welke momenten voor de afspraken én de operatie u uitkomen.

○	○	○	○	○
Onbelangrijk	Enigszins onbelangrijk	Neutraal	Enigszins belangrijk	Erg belangrijk

10. De wachttijd tot de operatie is zo kort mogelijk.

AMC
Meibergdreef 9
1105 AZ Amsterdam
Postbus 22660
1100 DD Amsterdam
T +31(0)20 566 9111
www.amc.nl

Onbelangrijk	Enigszins onbelangrijk	Neutraal	Enigszins belangrijk	Erg belangrijk
○	○	○	○	○

11. Artsen zijn makkelijk te bereiken voor vragen tijdens de wachttijd.

Onbelangrijk	Enigszins onbelangrijk	Neutraal	Enigszins belangrijk	Erg belangrijk
○	○	○	○	○

Kies van dezelfde uitspraken de <u>5 uitspraken</u> die u het belangrijkst vindt: wat moet het ziekenhuis écht regelen om goede zorg te leveren? Zet deze 5 uitspraken vervolgens op volgorde van wat u het belangrijkst vindt. Vul daarvoor de getallen 1 tot en met 5 in de open vakjes achter de stellingen. Geef daarvoor de stelling die u het ALLERBELANGRIJKST vindt een 1 en de stelling die u het MINST BELANGRIJK vindt een 5.

Bijvoorbeeld: u vindt 'artsen zijn makkelijk te bereiken voor vragen' het allerbelangrijkst. Dan zet u achter deze zin in het open vakje een 1. Daarna vindt u het belangrijk dat de operatiedatum snel bekend is: achter deze zin komt in het open vakje een 2. Zo gaat u door <u>tot het cijfer 5 is ingevuld</u>. Niet alle uitspraken krijgen dus een cijfer.

De zorgverleners houden rekening met uw wensen en verwachtingen.	
U heeft de afspraken in het ziekenhuis én de operatie bij telkens dezelfde arts.	
De informatie over de ziekte, afspraken en operatie is compleet en duidelijk.	
De operatiedatum is snel bekend.	
Er is voldoende aandacht voor lichamelijke klachten.	
Er is voldoende aandacht voor uw gevoelens en emoties.	
U krijgt voldoende informatie over wat de volgende stappen in de behandeling zijn.	
Het ziekenhuis houdt zich aan gemaakte afspraken en deze worden niet meer veranderd.	
U kunt zelf aangeven welke momenten voor de afspraken én de operatie u uitkomen.	
De wachttijd tot de operatie is zo kort mogelijk.	
Artsen zijn makkelijk te bereiken voor vragen.	

AMC
Meibergdreef 9
1105 AZ Amsterdam

Postbus 22660
1100 DD Amsterdam

T +31(0)20 566 9111
www.amc.nl

Is er nog iets wat hierboven niet is genoemd, maar waarvan het voor u heel belangrijk is dat een ziekenhuis het regelt tijdens de wachttijd?

Onderdeel 4:

In dit laatste onderdeel willen we graag van u weten wat de kwaliteit van uw leven in de wachttijd was. Zo zijn we geïnteresseerd in hoe u zich lichamelijk en geestelijk voelde in de wachttijd tot de operatie. Ook willen we graag weten hoe u uw dagen vulde tijdens het wachten op de operatie. We vragen ook hoe deze periode voor uw omgeving was.

Geef van de volgende uitspraken aan in welke mate deze voor de periode tussen de eerste afspraak in het Amsterdam UMC en de operatie voor u van toepassing waren (stap D in het voorbeeld). Omcirkel het antwoord dat voor u van toepassing is.

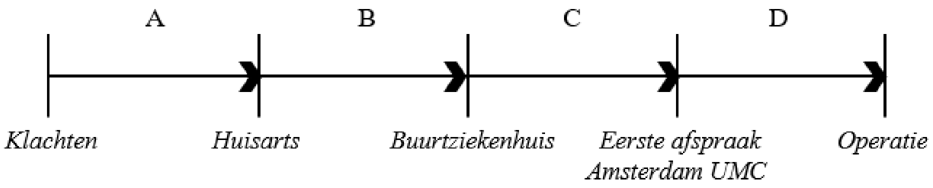

1. Ik voel me de laatste tijd gespannen.

   A. Meestal
   B. Vaak
   C. Af en toe, soms
   D. Helemaal niet

2. Ik geniet nog steeds van de dingen waar ik vroeger van genoot.

   A. Zeker zo veel
   B. Wat minder
   C. Duidelijk minder
   D. Nauwelijks nog

3. Ik krijg de laatste tijd het angstige gevoel alsof er elk moment iets vreselijks zal gebeuren.

   A. Heel zeker en vrij erg
   B. Ja, maar niet zo erg
   C. Een beetje, maar ik maak me er geen zorgen over
   D. Helemaal niet

4. Ik kan lachten en dingen van de vrolijke kant zien.

   A. Net zoveel als vroeger
   B. Nu wat minder
   C. Nu duidelijk minder
   D. Helemaal niet meer

5. Ik maak me de laatste tijd ongerust.

   A. Heel erg vaak
   B. Vaak
   C. Niet zo vaak
   D. Heel soms

6. Ik voel me de laatste tijd opgewekt.

   A. Helemaal niet
   B. Niet vaak
   C. Soms
   D. Meestal

7. Ik kan de laatste tijd rustig zitten en me ontspannen.

   A. Zeker
   B. Meestal
   C. Niet vaak
   D. Helemaal niet

8. Ik voel me de laatste tijd alsof alles moeizamer gaat.

   A. Bijna altijd
   B. Heel vaak
   C. Soms
   D. Helemaal niet

9. Ik krijg de laatste tijd een soort benauwd, gespannen gevoel in mijn maag.

   A. Helemaal niet
   B. Soms
   C. Vrij vaak
   D. Heel vaak

10. Ik heb de laatste tijd geen interesse meer in mijn uiterlijk.

    A. Zeker
    B. Niet meer zoveel als ik zou moeten
    C. Mogelijk wat minder
    D. Evenveel interesse als voorheen

11. Ik voel me de laatste tijd rusteloos.

    A. Heel erg
    B. Tamelijk veel
    C. Niet erg veel
    D. Helemaal

12. Ik verheug me van tevoren al op dingen.

    A. Net zoveel als vroeger
    B. Een beetje minder dan vroeger
    C. Zeker minder dan vroeger
    D. Bijna nooit

13. Ik krijg de laatste tijd plotseling gevoelens van angst of paniek.

    A. Zeer vaak
    B. Tamelijk vaak
    C. Niet erg vaak
    D. Helemaal niet

14. Ik kan van een goed boek genieten of een radio- of televisieprogramma.

    A. Vaak
    B. Soms
    C. Niet vaak
    D. Zelden

<u>Vragen over het effect van het wachten op uw directe omgeving</u>

15. Voor zover ik kan inschatten, voelen mensen in mijn directe omgeving zich de laatste tijd gespannen als gevolg van het wachten op de operatie

    A. Meestal
    B. Vaak
    C. Af en toe, soms
    D. Helemaal niet

16. Voor zover ik kan inschatten, voelen mensen in mijn directe omgeving zich de laatste tijd angstig of verdrietig als gevolg van het wachten op de operatie.

    A. Meestal
    B. Vaak
    C. Af en toe, soms
    D. Helemaal niet

<u>Vragen over lichamelijke klachten tijdens de wachttijd</u>

17. Tijdens de wachttijd gold ten aanzien van het slapen:

    A. U sliep net zoveel als voor de wachttijd
    B. U sliep iets minder als voor de wachttijd
    C. U sliep veel minder dan voor de wachttijd
    D. U gebruikte slaapmedicatie

18. Hoe vaak had u last van pijnklachten in de periode tussen de eerste afspraak in dit ziekenhuis en de operatie?

(Bij 'helemaal niet' als antwoord, ga direct naar vraag 21)

    A. Meestal
    B. Vaak
    C. Af en toe, soms
    D. Helemaal niet

19. Hoe hevig was uw pijn <u>gemiddeld</u> in de periode tussen de eerste afspraak in dit ziekenhuis en de operatie? Plaats op de volgende pagina een streep van boven naar beneden op de lijn die het best de ernst van uw pijn aangeeft. Zoals hier:

Voorbeeld

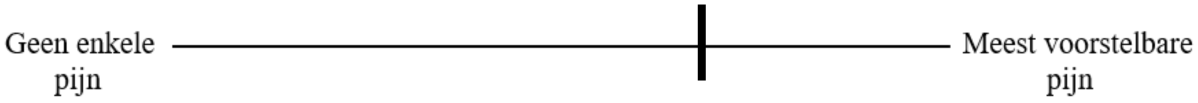

Plaats nu hieronder zelf een streep op de lijn die het best de ernst van uw pijn aangeeft:

Geen enkele pijn ———————————————————————— Meest voorstelbare pijn

20. Hoe hevig was uw pijn <u>op de slechtste momenten</u> in de periode tussen de eerste afspraak in dit ziekenhuis en de operatie? Plaats weer een streep van boven naar beneden op de lijn die het best de ernst van uw pijn aangeeft.

Geen enkele pijn ———————————————————————— Meest voorstelbare pijn

21. Had u last van lichamelijke klachten, anders dan pijnklachten, in de periode tussen de eerste afspraak in dit ziekenhuis en de operatie?

(Bij 'nee' als antwoord, ga direct naar vraag 23)

A. Ja
B. Nee

22. Van welke lichamelijke klachten, anders dan pijnklachten, had u last in de periode tussen de eerste afspraak in het ziekenhuis en de operatie?

(U mag meerdere opties omcirkelen)

A. Vaginaal bloedverlies
B. Buikpijn, misselijkheid of diarree
C. Klachten bij het plassen
D. Moeheid, futloos gevoel
E. Overig:____

AMC
Meibergdreef 9
1105 AZ Amsterdam
Postbus 22660
1100 DD Amsterdam
T +31(0)20 566 9111
www.amc.nl

23. Hadden deze pijnklachten of andere lichamelijke klachten invloed op uw dagelijkse bezigheden?

    A. Heel erg
    B. Tamelijk veel
    C. Niet erg veel
    D. Helemaal niet
    E. Niet van toepassing

<u>Vragen over activiteiten tijdens de wachttijd</u>

24. In de tijd dat ik moest wachten op mijn operatie deed ik ongeveer dezelfde dingen als voor die tijd

    A. Eens
    B. Enigszins eens
    C. Enigszins oneens
    D. Oneens

25. In de wachttijd ben ik minder tijd gaan besteden aan de volgende dingen:

(U mag hier meerdere opties omcirkelen)

    A. Werk (betaald- of vrijwilligerswerk)
    B. Studie
    C. Zorgen voor partner / kinderen of andere mensen
    D. Bewegen of sporten
    E. Hobby
    F. Ontspanning
    G. Huishouden
    H. Tijd met vrienden/familie
    I. Informatie opzoeken over ziekte of operatie
    J. Overig:____

AMC
Meibergdreef 9
1105 AZ Amsterdam
Postbus 22660
1100 DD Amsterdam
T +31(0)20 566 9111
www.amc.nl

26. In de wachttijd ben ik meer tijd gaan besteden aan de volgende dingen:

(U mag hier meerdere opties omcirkelen)

A. Werk
B. Studie
C. Zorgen voor partner / kinderen of andere mensen
D. Bewegen
E. Hobby
F. Ontspanning
G. Huishouden
H. Tijd met vrienden / familie
I. Informatie opzoeken over ziekte of operatie
J. Overig:

27. Werkte u (betaald werk of vrijwilligerswerk buitenshuis) voordat u wist dat u geopereerd moest worden?

(Bij "nee" als antwoord, ga direct naar vraag 30)

A. Ja, betaald werk buitenshuis
B. Ja, vrijwilligerswerk buitenshuis
C. Ja, beide
D. Nee

28. U heeft aangegeven dat u werkte (betaald werk of vrijwilligerswerk buitenshuis). In de tijd dat u moest wachten op de operatie hoeveel heeft u in deze periode gewerkt?

A. Net zoveel als vroeger
B. Een beetje minder dan vroeger
C. Zeker minder dan vroeger
D. Bijna niet

29. Indien u MINDER of NIET gewerkt heeft in de periode tussen de eerste afspraak in dit ziekenhuis en de operatie: welke reden had dit?

(U kunt meerdere antwoorden omcirkelen)

A. Op advies van arbo-arts
B. Op advies van een andere arts
C. Omdat u lichamelijke klachten door de ziekte had
D. Omdat u emotionele of geestelijke klachten door de ziekte had
E. Op advies van andere uit uw omgeving
F. Overig:____
G. Niet van toepassing

AMC
Meibergdreef 9
1105 AZ Amsterdam
Postbus 22660
1100 DD Amsterdam
T +31(0)20 566 9111
www.amc.nl

30. Als u kijkt naar de mogelijke stappen in de wachttijd: welk onderdeel heeft u als het MEEST vervelend ervaren?

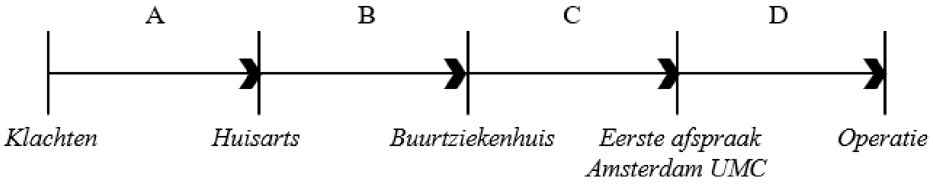

Het MEEST vervelend was de tijd tussen:

A. Het hebben van klachten en de afspraak bij de huisarts
B. De verwijzing vanuit de huisarts naar het buurtziekenhuis
C. De verwijzing vanuit het buurtziekenhuis naar het Amsterdam UMC
D. De eerste afspraak in het Amsterdam UMC en de operatie
E. Niet van toepassing

Hartelijk dank voor het meewerken aan dit onderzoek!

Heeft u vragen?
Bij vragen kunt u deze stellen aan uw behandelend gynaecoloog of contact opnemen met het onderzoeksteam.

<u>Contact gegevens onderzoeksteam:</u>
Amsterdam UMC, locatie AMC
Afdeling Gynaecologie & Verloskunde
Email: j.w.m.aarts@amsterdamumc.nl
