# Supplementary material for: Patients’ preferences, experiences and expectations with wait time until surgery in gynaecological oncology: a mixed-methods study in two gynaecological oncological centres in the Netherlands
Source: BMJ Open. 2024 Aug 17;14(8):e085932. doi: 10.1136/bmjopen-2024-085932 (PMC11331850; doi:10.1136/bmjopen-2024-085932)
Supplement: Supplementary file 4 [file bmjopen-2024-085932.reviewer_comments.pdf]

PEER REVIEW HISTORY

BMJ Open publishes all reviews undertaken for accepted manuscripts. Reviewers are asked to complete a checklist review form and are provided with free text boxes to elaborate on their assessment. These free text comments are reproduced below.

ARTICLE DETAILS

**TITLE (PROVISIONAL)**	Patients' preferences, experiences and expectations with wait time until surgery in gynaecological oncology: a mixed-methods study in two gynaecological oncological centres in The Netherlands.
**AUTHORS**	van den Berg, Kim; Knegt, Anne; Fons, Guus; Lok, Christianne; Aarts, Johanna W.M.

VERSION 1 - REVIEW

**REVIEWER NAME**	***Jordan M Alpert***
**REVIEWER AFFILIATION**	Cleveland Clinic
**REVIEWER CONFLICT OF INTEREST**	none
**DATE REVIEW RETURNED**	22-Apr-2024

**GENERAL COMMENTS**	This study analyzed survey results among patients with cancer after surgery was performed. The goal was to understand the impact of waiting for the surgery. It seems as if valuable qualitative data was collected, but none is reported. The survey results report that wait times are undesirable, but the importance of the results are sometimes overstated. Below are additional comments/questions:<br><br>Introduction<br>- Define patient-centredness. It has various definitions, so clarifying how you're conceptualizing it would be helpful to readers.<br>- Line 13-20, the benefit of patient centered care on gynaecological oncological care is unclear. It is associated with high emotional impact and intensive treatments? This needs clarification.<br>- There is an opportunity to include anxiety associated with wait times, as well as general depression that can occur from a cancer diagnosis in the introduction, which would strengthen the rationale for conducting this study.<br><br>Methods<br>- Exclusion and inclusion criteria can be listed more succinctly. Almost every sentence begins with "We excluded…"<br>- Results are included in the methods section. These should be moved to the results section. All the text beginning on line 57, "This resulted in a list of themes…" should be moved.<br>- How was the semi-structured questionnaire constructed? How was the codebook formed? What was the process of generating themes? What occurred to indicate that consensus was reached? This type of additional detail is needed.<br>- The study describes an exploratory sequential mixed methods design. I'd encourage the authors to use this terminology and structure the methods appropriately.<br>- How were patients approached/contacted to participate?<br><br>Results

	- Were patients spending less time doing physical activities during the wait time because they couldn't physically do those activities, or because of the anxiety of the upcoming operation?<br>- What was the rationale for comparing the data based on educational level in the regression analysis? Is it hypothesized that those with higher education would have a better understanding of the need to wait longer? Comparing patients based on their stage of cancer might be more interesting.<br>- No qualitative results are reported. I understand that the qualitative portion may have only served to assist with survey development, but if 11 themes were generated, that seems like important data to report. If the qualitative results were so minor that their only use was to guide the survey, then I wouldn't call this a mixed methods study and devote space to describe the qualitative analysis. Instead, the qualitative portion should be minimized and mentioned almost as a footnote, since the quantitative analysis is the centerpiece of the study.<br>- How were questions on the survey worded about the impact of wait times? It's possible patients would have anxiety, depression, pain, trouble sleeping no matter the timing of the surgery. In addition, patients scheduled to have surgery the next day (even if they've already waited 5 weeks) might be experiencing more of those factors than a patient 3 weeks out.<br><br>Discussion<br>- It might be an overstatement to claim that waiting time affects quality of life. There are so many confounding factors that contribute to QoL, making it difficult to isolate wait times as the reason.<br>- The authors state there are a number of ways patient-centeredness can be improved, but only wait times are discussed. Wait times are not a factor of patient-centeredness. They are related to patient-centeredness, but communication, listening, involvement, empathy…etc. are the factors that should be considered and explained when it comes to wait times.<br>- It's problematic to mention results of interviews when no interview results are included (line 17)<br>- More of the discussion should focus on the diagnosis. Patients experience anxiety because they are worried they may die or their cancer will spread, unless the surgery happens in a timely manner. They're not necessarily worried about the wait time, but the consequences of the wait time. As the authors discuss, there can be benefits to waiting (i.e. planning), so casting wait times as always negative does not capture this phenomenon.

**REVIEWER NAME**	***fan xu***
**REVIEWER AFFILIATION**	Nanchong central hospital
**REVIEWER CONFLICT OF INTEREST**	We declared no conflict of interest with the authors.
**DATE REVIEW RETURNED**	12-May-2024

**GENERAL COMMENTS**	In the manuscript entitled "Patients' preferences, experiences and expectations with waiting time until surgery in gynaecological oncology: a mixed-methods study, " van den Berg et al. examined the physical and mental states of patients with gynecological malignancies from their own perspective while awaiting surgery. This study presents some interesting findings that are invaluable for enhancing patient-centered care.

	#1 On page 3, line 30, what is the threshold referred to here?<br>#2 On page 3, lines 32-41, could you please include the 95% confidence interval in the results section?<br>#3 Regarding Table 3 on page 14, lines 8-25, I am interested in knowing what the incidence of positive events is, as well as the criteria for selecting the variables included in the multivariate regression analysis.<br>#4 In this study, how did the authors evaluate the patient's quality of life? Was a self-made scale used? Have they also considered well-established quality of life scales, such as the EQ-5D or SF-36?

**REVIEWER NAME**	***Alexandra Lamacki***
**REVIEWER AFFILIATION**	University of Chicago Division of the Biological Sciences, Obstetrics and Gynecology
**REVIEWER CONFLICT OF INTEREST**	I confirm that I have no competing interests.
**DATE REVIEW RETURNED**	18-May-2024

**GENERAL COMMENTS**	Overall this is an important piece of research for healthcare providers. It provides insights into an often ignored period of time - the waiting period between the plan of car and the treatment initiation. Despite its setting in a national healthcare system that is distinct from that of other countries (particularly the U.S.), their conclusions have relevance to all healthcare systems. I recommend publication of this paper after satisfactory minor revisions.<br><br>Abstract:<br>1) The first sentence of the “Objectives” portion (line 6) could be stronger and more specific to the research being presented. “There seems room for” does not immediately convince the reader that this is important work for patient-centered care.<br>2) The first sentence of the “Design and setting” portion (line 16) should be a full sentence.<br><br>Introduction:<br>3) On page 5 line 27: I recommend providing a citation for rising cancer incidences and the increased financial/personnel burden to the healthcare system.<br>4) Are wait times still affected by COVID-19? Or have they gone back to pre-pandemic levels?<br>5) Regarding page 5, line 43: Can you specify which bodies have created these guidelines on decision-to-treatment timing?<br><br>Methods:<br>6) Please provide further information about the consenting process for participants both for the interviews and the survey.<br>7) The section title of “1. Semi-structured Interviews” (line 47) should be capitalized.<br>8) Do not include your results in the Methods section such as the second half of the paragraph following “1. Semi-structured Interviews.” Reserve this information for the results section and focus on methodology here.<br>9) What do you mean by “self-developed questions”? (line 35 on page 8)<br>10) Regarding the definition of the dependent variable of wait time as a dichotomous variable: I’m assuming from your description that you had three options in your survey, but you analyzed it as a dichotomous variable because only two responses were selected by

	patients. I would just clarify in your description what options were present in the survey.<br>11) I recommend including the questionnaire in the supplementary material so that others could utilize it in the future.<br><br>Results:<br>12) For Table 1: The description of the "a" superscript is missing a word – "Stage was classified as undefined if there was…"<br>13) Consider being consistent with how you report time, i.e., only reporting in days instead of weeks and then converting to days.<br>14) On page 14, line 58: Do you mean work force challenges/resource constraints in the healthcare field, or for the patients?<br>15) On page 14 line 60: I don't understand what you mean by "Given constant resources, it would imply that other patients would have to wait longer." What does "constant resources" refer to, and do you mean patients getting other types of procedures? Please clarify.<br>16) I would be cautious about stating that female sex is related to higher levels of emotional distress (page 15 line 60). Even if there is some statistic that can be cited, we must recognize that studies can have biases, and we should not propagate these biases.<br><br>Strengths and limitations:<br>17) Well-stated strengths and limitations<br><br>Future directions:<br>18) Future directions could also include studies on healthcare efficiency and how to improve wait times as well as improving patient education regarding their diagnoses and preparing for surgery to decrease the emotional burden.<br><br>Conclusion:<br>19) Well stated conclusion.

VERSION 1 – AUTHOR RESPONSE

Reviewer 1 Alpert

1. *This study analyzed survey results among patients with cancer after surgery was performed. The goal was to understand the impact of waiting for the surgery. It seems as if valuable qualitative data was collected, but none is reported. The survey results report that wait times are undesirable, but the importance of the results are sometimes overstated. Below are additional comments/questions:*
   Thank you for your valuable feedback. We have added a section with the qualitative results from the interviews, and we have nuanced the claims about conclusions to be drawn from the quantitative part.
2. *Define patient-centredness. It has various definitions, so clarifying how you're conceptualizing it would be helpful to readers.*
   We added a definition with reference.
3. *Line 13-20, the benefit of patient centered care on gynaecological oncological care is unclear. It is associated with high emotional impact and intensive treatments? This needs clarification*
   This has been changed to clarify in conjunction with the next point.
4. *There is an opportunity to include anxiety associated with wait times, as well as general depression that can occur from a cancer diagnosis in the introduction, which would strengthen the rationale for conducting this study*
   This is a good point, we have added to our manuscript in the introduction to include this.

5. *Exclusion and inclusion criteria can be listed more succinctly. Almost every sentence begins with "We excluded…"*
   Text has been changed to make this more succinct.
6. *Results are included in the methods section. These should be moved to the results section. All the text beginning on line 57, "This resulted in a list of themes…" should be moved.*
   We have added more detail on the first, qualitative part of our study. We added a section to both methodology and results. This part was moved to the results section that contains the results from the interviews.
7. *How was the semi-structured questionnaire constructed? How was the codebook formed? What was the process of generating themes? What occurred to indicate that consensus was reached? This type of additional detail is needed.*
   We added a section to the Methods to address these questions.
8. *The study describes an exploratory sequential mixed methods design. I'd encourage the authors to use this terminology and structure the methods appropriately.*
   We have used this terminology now and structured the methods accordingly.
9. *How were patients approached/contacted to participate?*
   We regret that we failed on describing this in the first version. We now added details of how patients were contacted and recruited in the Methods section.
10. *Were patients spending less time doing physical activities during the wait time because they couldn't physically do those activities, or because of the anxiety of the upcoming operation?*
    We did not ask this specifically in the questionnaire so unfortunately are not able to answer this based on this study. It is however a very relevant question and something for a follow up study.
11. *What was the rationale for comparing the data based on educational level in the regression analysis? Is it hypothesized that those with higher education would have a better understanding of the need to wait longer? Comparing patients based on their stage of cancer might be more interesting.*
    We hypothesized that education level might result in differences in finding the way to the hospital in case of anxiety or worry and that differences in understanding might also result in differences in acceptance of wait time. Disease stage was also a variable which was analysed (see methods). This was, however, not significantly correlated with the independent variables.
12. *No qualitative results are reported. I understand that the qualitative portion may have only served to assist with survey development, but if 11 themes were generated, that seems like important data to report. If the qualitative results were so minor that their only use was to guide the survey, then I wouldn't call this a mixed methods study and devote space to describe the qualitative analysis. Instead, the qualitative portion should be minimized and mentioned almost as a footnote, since the quantitative analysis is the centerpiece of the study*
    Thank you for this feedback. We added a section in Methods and results about the qualitative data that we gathered. We agree that this add more depth to the results.
13. *How were questions on the survey worded about the impact of wait times? It's possible patients would have anxiety, depression, pain, trouble sleeping no matter the timing of the surgery. In addition, patients scheduled to have surgery the next day (even if they've already waited 5 weeks) might be experiencing more of those factors than a patient 3 weeks out.*
    The survey was administered during or shortly after admission in all patients so there were no big differences between patients in this respect. We administered a validated questionnaire about anxiety and depression in the hospital setting. We did not specifically ask them if they were anxious because of wait time but rather how they scored on this scale of anxiety and depression.
14. *It might be an overstatement to claim that waiting time affects quality of life. There are so many confounding factors that contribute to QoL, making it difficult to isolate wait times as the reason.*
    We did not intend to imply causality, but rather indicate that many aspects of QoL are impacted during wait time. Discussion was altered accordingly.
15. *The authors state there are a number of ways patient-centeredness can be improved, but only wait times are discussed. Wait times are not a factor of patient-centeredness. They are*

related to patient-centeredness, but communication, listening, involvement, empathy…etc. are the factors that should be considered and explained when it comes to wait times.
We have also included the aspects of QoL that we analyzed that were negatively impacted. We have tried to make this text clearer.

16. *It's problematic to mention results of interviews when no interview results are included (line 17)*
    We have now included results from the interviews. See our comments above.
17. *More of the discussion should focus on the diagnosis. Patients experience anxiety because they are worried they may die or their cancer will spread, unless the surgery happens in a timely manner. They're not necessarily worried about the wait time, but the consequences of the wait time. As the authors discuss, there can be benefits to waiting (i.e. planning), so casting wait times as always negative does not capture this phenomenon.*
    We fully agree with this point and have changed the discussion to make this clearer. We have added this more clearly to the discussion.

Reviewer 2 Fan Xu

1. *In the manuscript entitled "Patients' preferences, experiences and expectations with waiting time until surgery in gynaecological oncology: a mixed-methods study, " van den Berg et al. examined the physical and mental states of patients with gynecological malignancies from their own perspective while awaiting surgery. This study presents some interesting findings that are invaluable for enhancing patient-centered care.*
   Thank you kindly for your compliments and feedback.
2. *On page 3, line 30, what is the threshold referred to here?*
   We added an explanation
3. *On page 3, lines 32-41, could you please include the 95% confidence interval in the results section?*
   We added the confidence intervals.
4. *Regarding Table 3 on page 14, lines 8-25, I am interested in knowing what the incidence of positive events is, as well as the criteria for selecting the variables included in the multivariate regression analysis.*
   Before performing the analyses we formulated hypotheses about factors that are correlated with wait time, acceptability of wait time and experience of wait time. We based these hypotheses on the limited available literature and on the results from the interviews. For example some interviewees described that because of the pain they experienced they felt that waiting was too long. Or some mentioned that because wait time was in line with their expectations they felt it was an acceptable period of time.
5. *In this study, how did the authors evaluate the patient's quality of life? Was a self-made scale used? Have they also considered well-established quality of life scales, such as the EQ-5D or SF-36?*
   We used a validated questionnaire on anxiety and depression. Other than that we determined aspects of patients' lives that were impacted during wait time in the interviews and then based the questionnaire on this. We elaborated on this in the methods section to make this clearer. We did not use the EQ-5D or SF-36,.This is a good suggestion for further research.

Reviewer 3 Lamacki

Overall this is an important piece of research for healthcare providers. It provides insights into an often ignored period of time - the waiting period between the plan of car and the treatment initiation. Despite its setting in a national healthcare system that is distinct from that of other countries (particularly the U.S.), their conclusions have relevance to all healthcare systems. I recommend publication of this paper after satisfactory minor revisions.

Thank you for your feedback and compliments.

1. *The first sentence of the "Objectives" portion (line 6) could be stronger and more specific to the research being presented. "There seems room for" does not immediately convince the reader that this is important work for patient-centered care.*
   This was changed to make it stronger and more specific.
2. *The first sentence of the "Design and setting" portion (line 16) should be a full sentence.*
   This was changed.
3. *On page 5 line 27: I recommend providing a citation for rising cancer incidences and the increased financial/personnel burden to the healthcare system.*
   This was added
4. *Are wait times still affected by COVID-19? Or have they gone back to pre-pandemic levels?*
   We do notice that wait times are trending upwards, for oncology patients this tends to be limited as patients tend to be given (some degree of) priority in planning. Staff shortages seem to be the main driver here, we have added observations to this effect. See the Introduction section.
5. *Regarding page 5, line 43: Can you specify which bodies have created these guidelines on decision-to-treatment timing?*
   This was added.
6. *Please provide further information about the consenting process for participants both for the interviews and the survey.*
   See also our comment to the reviewer above. We have added more detail about this process to the Methods section.
7. *The section title of "1. Semi-structured Interviews" (line 47) should be capitalized.*
   This was changed
8. *Do not include your results in the Methods section such as the second half of the paragraph following "1. Semi-structured Interviews." Reserve this information for the results section and focus on methodology here.*
   This was changed as we re-arranged the methds and results section to add the qualitative part presenting the results from the interviews.
9. *What do you mean by "self-developed questions"? (line 35 on page 8)*
   We deleted this phrase. We clarified in the methods section how we developed the questionnaire.
10. *Regarding the definition of the dependent variable of wait time as a dichotomous variable: I'm assuming from your description that you had three options in your survey, but you analyzed it as a dichotomous variable because only two responses were selected by patients. I would just clarify in your description what options were present in the survey.*
    This was clarified.
11. *I recommend including the questionnaire in the supplementary material so that others could utilize it in the future.*
    We added the questionnaire
12. *For Table 1: The description of the "a" superscript is missing a word – "Stage was classified as undefined if there was…"*
    This was changed
13. *Consider being consistent with how you report time, i.e., only reporting in days instead of weeks and then converting to days.*
    We have changed this.
14. *On page 14, line 58: Do you mean work force challenges/resource constraints in the healthcare field, or for the patients?*
    We meant work force challenges / resource constraints, and we have changed the wording to clarify this.
15. *On page 14 line 60: I don't understand what you mean by "Given constant resources, it would imply that other patients would have to wait longer." What does "constant resources" refer to, and do you mean patients getting other types of procedures? Please clarify.*
    We understand this was not clear. We have changed the wording to reflect that we mean that if cancer patients are given preference, with the same amount of staff / surgical time / resources this would mean that non-oncological patients would have to wait longer.

16. *I would be cautious about stating that female sex is related to higher levels of emotional distress (page 15 line 60). Even if there is some statistic that can be cited, we must recognize that studies can have biases, and we should not propagate these biases.*
    We have removed this. Thank you.
17. *Well-stated strengths and limitations*
    Thank you!
18. *Future directions could also include studies on healthcare efficiency and how to improve wait times as well as improving patient education regarding their diagnoses and preparing for surgery to decrease the emotional burden.*
    This is a good point, we have changed the future directions part and included this suggestion
19. *Well stated conclusion.*
    Thank you for your compliments.

VERSION 2 – REVIEW

**REVIEWER NAME**	***Jordan M Alpert***
**REVIEWER AFFILIATION**	Cleveland Clinic
**REVIEWER CONFLICT OF INTEREST**	none
**DATE REVIEW RETURNED**	17-Jul-2024

**GENERAL COMMENTS**	Thank you for addressing my comments.

**REVIEWER NAME**	***fan xu***
**REVIEWER AFFILIATION**	Nanchong central hospital
**REVIEWER CONFLICT OF INTEREST**	We conclude that there were no conflicts of interest.
**DATE REVIEW RETURNED**	29-Jul-2024

**GENERAL COMMENTS**	In the manuscript titled "Patients’ Preferences, Experiences, and Expectations Regarding Waiting Time for Surgery in Gynaecological Oncology: A Mixed-Methods Study", van den Berg et al. investigated the physical and psychological conditions of patients with gynecological malignancies from the patients' own perspectives during the preoperative waiting period. The study yields significant findings that are crucial for advancing patient-centered care. The revisions made to this article are deemed satisfactory, and its publication is recommended.
